# Chitosan–silver nanocomposites: promising alternatives for combating azole-resistant *Aspergillus fumigatus*

**DOI:** 10.3389/fcimb.2025.1669609

**Published:** 2026-01-12

**Authors:** Tian Yu, Jianan Wu, Tingting Li, Yilin Zhu, Yanwei Xie, Zerui Yin, Youzhen Ma, Wenlong Du

**Affiliations:** 1 Department of Bioinformatics, School of Life Sciences, Xuzhou Medical University, Xuzhou, Jiangsu, China; 2 Department of Biophysics, School of Life Sciences, Xuzhou Medical University, Xuzhou, Jiangsu, China

**Keywords:** antifungal activity, *Aspergillus fumigatus*, chitosan, chitosan‑silver nanocomposites, silver nanoparticles, reactive oxygen species

## Abstract

**Background:**

The emergence of azole-resistant strains of *Aspergillus fumigatus* presents a significant challenge in clinical and settings, necessitating the development of alternative antifungal strategies. Chitosan-silver nanocomposites (Chi-AgNPs) have emerged as promising candidates due to their dual antimicrobial mechanisms and enhanced physicochemical properties.

**Objectives:**

This study aimed to evaluate the synthesis, characterization, antifungal properties, and low toxicity in an invertebrate model of Chi-AgNPs compared with those of chitosan and silver nanoparticles (AgNPs) alone, particularly against azole-resistant *A. fumigatus*.

**Methods:**

Chi-AgNPs were synthesized via a chemical reduction method and characterized using particle size analysis, ultraviolet–visible (UV–Vis) spectroscopy, Fourier transform infrared (FTIR), scanning electron microscopy (SEM), and transmission electron microscopy (TEM). Antifungal efficacy was assessed through minimum inhibitory concentration (MIC), minimum fungicidal concentration (MFC), microbial growth curves, and *in vivo* tests using *Galleria mellonella* larvae. Reactive oxygen species (ROS) generation and fungal cell wall deformation were analyzed to explore the antifungal mechanism.

**Results:**

Compared with chitosan and AgNPs alone, Chi-AgNPs exhibited superior physicochemical properties, with smaller and more uniform particle sizes (average: 212.5 nm) and improved dispersion stability based on immediate characterization data. The MIC and MFC of Chi-AgNPs (8–16 μg/mL and 128 μg/mL, respectively) were significantly lower than those of chitosan and AgNPs (128 μg/mL and >256 μg/mL, respectively). Compared with AgNPs, Chi-AgNPs induced higher ROS levels (*p* < 0.05) and were associated with more severe fungal cell wall damage. *In vivo*, the Chi-AgNPs-treated fungus-infected group presented the lowest mortality in *G. mellonella* larvae (*p* < 0.05), demonstrating superior antifungal activity and low toxicity in an invertebrate model.

**Conclusions:**

This study highlights the potential of Chi-AgNPs as promising alternatives to combat azole-resistant fungal infections. These findings provide a foundation for further development of Chi-AgNPs as novel antifungal agents with broad applicability.

## 1 Introduction

*A. fumigatus*, a filamentous fungus, is ubiquitously distributed across diverse geographical regions and can proliferate on a wide array of substrates (Van De Veerdonk et al., 2025). The conidium of *A. fumigatus* is physically spherical to subspherical, moderately sized and able to bypass mucociliary clearance to reach the lower respiratory tract. Its spores are widely distributed in the air and are easy for humans to access (Morrissey et al., 2024). The inhalation of conidia typically results in their rapid eradication in immunocompetent hosts. In contrast, immunocompromised individuals fail to eradicate the fungi, which may lead to severe invasive aspergillosis (IA) (Lionakis et al., 2023). The mortality rate associated with such infections exceeds 50%, with *A. fumigatus* being the most prevalent causative agent (Peláez-García de la Rasilla et al., 2023). Azole drugs (such as itraconazole, esaconazole, and voriconazole) are often used to treat IA and other serious fungal infections (Fueyo Álvarez et al., 2025; Zhu et al., 2025). Antifungal agents have been extensively utilized in clinical practices, and their misuse has resulted in increased fungal tolerance to these drugs, contributing to the emergence of numerous drug-resistant strains (Burks et al., 2021). Therefore, there is an urgent need to develop new antifungal therapies to address infections caused by these resistant strains (Zhu et al., 2023).

Chitosan is a commonly used biopolymer that is obtained from chitosan through industrial deacetylation (Dambuza et al., 2025). It is commonly used as a reducing agent and stabilizer because of its good biocompatibility, biodegradability, nonallergenic nature, nontoxicity, and low price (Nahórny et al., 2022). Chitosan is composed of two amino and hydroxyl functional groups, which can be combined with metal ions, so it can also be used as a cationic surfactant to modify metal nanomaterials (Lu et al., 2025). When chitosan is conjugated with a metal, the resulting nanocomposites exhibit antimicrobial properties. This process not only significantly enhances the natural antimicrobial effects of chitosan but also opens up great possibilities for more fine-tuning the structure and morphology of metal oxides, a technique also known as biomineralization (Nahórny et al., 2022; Wang et al., 2022). In addition, chitosan, a biodegradable and biocompatible polysaccharide derived from chitin, has inherent antimicrobial properties due to its ability to interact with negatively charged microbial cell walls, leading to increased membrane permeability and cell death (Rashki et al., 2021; Gao and Wu, 2022), and chitosan-coated crops also have certain antimicrobial effects on fungi (Ke et al., 2022). However, the poor barrier ability, poor antioxidant activity and limited antimicrobial properties of chitosan limit its application.

Nanotechnology has shown potential in combatting fungal infections (Paul and Hakkimane, 2025). Owing to their unique mode of action, smaller size, and higher surface-to-volume ratio, nanoparticles (NPs) are used to target fungi. This enables NPs to carry specific bioactive drugs to the target (Talank et al., 2022). Among all kinds of nanoparticles, metal oxide nanoparticles have attracted much attention because of their unique properties. There are different categories of metal NPs, including gold, silver, zinc, copper, and iron (Atik et al., 2025; Sharma et al., 2025). Smaller sizes, higher surface-to-volume ratios, surface clipping properties, and the ability to capture drug payloads may enable drug delivery at target sites. Damage to human cells caused by fungi can be reduced by treating fungi with nanomedicine preparations. Pharmaceutical nanoparticle formulations using nanotechnology are an important way to develop potential therapeutic interventions against microbial infections. AgNPs have good antimicrobial effects (Gad El-Rab et al., 2021; Gaikwad et al., 2024) and high stability and have not been proven to cause drug resistance; however, the use of AgNPs is often limited by their tendency to aggregate in solution, which reduces their bioavailability and antimicrobial efficacy (He et al., 2022). To address this limitation, natural polymers such as chitosan have been employed as stabilizing and functionalizing agents for silver nanoparticles (Balciunaitiene et al., 2024; De Moraes Barbosa Côrtes-Bogniotti et al., 2025).

Despite the promising potential of Chi-AgNPs nanocomposites, relatively few studies have explored their antifungal properties against azole-resistant strains of *A. fumigatus*. Previous research has focused primarily on bacterial pathogens or non-resistant fungal models (Artunduaga Bonilla et al., 2021; Shehabeldine et al., 2022; Radan et al., 2025), leaving a significant gap in understanding the potential of Chi-AgNPs against azole-resistant fungi. Furthermore, while the mechanisms underlying the antimicrobial activity of AgNPs and chitosan have been extensively studied (Tang et al., 2023), the enhanced and potentially synergistic antifungal mechanisms of Chi-AgNPs compared to their individual components—particularly their ability to induce ROS-mediated fungal cell damage in resistant strains—remain poorly understood.

This study aims to fill this gap by providing a direct comparative analysis of chitosan, AgNPs, and Chi-AgNPs against azole-resistant *A. fumigatus*. The physicochemical characteristics were evaluated via particle size analysis, UV–visible spectroscopy, FTIR, SEM, and TEM to confirm the successful integration of silver nanoparticles with chitosan and their dispersion stability. The antifungal properties of Chi-AgNPs were assessed both *in vitro* and *in vivo* against wild-type and azole-resistant strains of *A. fumigatus*, and the mechanisms of action were explored through ROS generation and fungal cell wall deformation. Additionally, low toxicity in an invertebrate model determined the potential applicability of Chi-AgNPs in clinical settings.

## 2 Materials and methods

### 2.1 Reagents

Chitosan was procured from Shanghai Macklin Biochemical Co., Ltd. (China). AgNPs were procured from Shanghai Win Pharmaceutical Technology Co., Ltd. (China). Argentum nitricum was obtained from Sinopharm Group Chemical Reagent Co., Ltd. (China). Resazurin was obtained from Shanghai Macklin Biochemical Co., Ltd. (China). Phosphate-buffered saline (PBS) was acquired from Jiangsu Keygen Biotech Co., Ltd. (China).

### 2.2 Preparation of strains

This study examined four strains: the wild-type *A. fumigatus* 1161 and the azole-resistant strains ED-22G, ED-23G (Chen et al., 2024) (isolated from the environment), Shjt40, and Shjt42b (isolated from clinical patients) (Du et al., 2021, Du et al., 2023; Hong et al., 2025). The clinical isolates Shjt40 and Shjt42b were obtained from a de-identified strain bank and their use was in compliance with ethical guidelines. *A. fumigatus* was preserved at -20°C in 50% glycerol. Unless otherwise stated, the concentration of fungal suspensions utilized in subsequent experiments was 1×10^6^ CFU/mL.

### 2.3 Synthesis of chitosan

Using an analytical balance with an accuracy of 1/10,000, 0.2048 g of chitosan was weighed and dissolved in 200.8 mL of sterile water. A total of 2.5 mL of glacial acetic acid and 1.5 mL of glycerol were added, the mixture was stirred thoroughly at 40°C with a magnetic stirrer for 2 h (Yuan et al., 2023), and a chitosan solution of 2048 μg/mL was successfully prepared.

### 2.4 Synthesis of AgNPs

The stable AgNPs mixture was prepared with glycerol as the base solution and ammonium citrate as the dispersant (Ghasemi and Jalal, 2016). The AgNPs (5.12 mg) and ammonium citrate (5.12 mg) were weighed and dissolved in 10.0 mL of glycerol via a 1/10,000 analytical balance. The mixture was stirred thoroughly at room temperature for 6 h via a magnetic stirrer, and a 512 μg/ml AgNPs mixture was prepared successfully. To maintain its stability and avoid the influence of light, the solution was treated without light, properly dried and stored for subsequent experiments.

### 2.5 Synthesis of chitosan–silver nanocomposites

Chi-AgNPs were prepared via a chemical reduction method with chitosan as the reducing agent and stabilizer. A total of 0.1275 g of silver nitrate and 90 mg of chitosan were weighed with a 1/10,000 analytical balance. The mixture was dissolved in 93.75 mL of water, and 0.9 mL of glacial acetic acid was added. Next, 150 μL of sodium hydroxide was added dropwise to the mixture of chitosan and silver nitrate under continuous stirring to ensure controlled reduction and stabilization. The mixture was stirred thoroughly at 90°C with a magnetic stirrer for 6 h (Dara et al., 2020). The solution changed from colorless to light yellow brown, indicating that a 1345 μg/ml chitosan-silver nanocomposites solution was successfully prepared. To maintain its stability and avoid the influence of light, the solution was protected from light, properly dried and stored for subsequent experiments.

### 2.6 Characterization of the nanocomposites

#### 2.6.1 Particle size of the nanocomposites

To determine the size distribution of the nanocomposite particles, the right amount of solution was taken from the sample, and a particle size analyzer (NICOMP 380NLS) was used for accurate particle size determination.

#### 2.6.2 Ultraviolet–visible spectral absorption analysis

Appropriate amounts of three sample solutions were placed in cuvettes, with distilled water first used as a blank for correction, followed by spectral measurements via a UV–Vis spectrophotometer (UV1800PC) within the range of 300–600 nm.

#### 2.6.3 Fourier transform infrared spectroscopy analysis

An appropriate amount of sample mixture was subjected to rotary evaporation (60°C), followed by vacuum drying at 50°C for 2 hours to obtain dried sample powder. FTIR spectroscopy was subsequently employed with a resolution of 4 cm⁻¹ allowing detection of shifts greater than 8 cm⁻¹ as significant and 32 scans per sample for infrared spectral characterization of the powder.

#### 2.6.4 Scanning electron microscopy analysis

Scanning electron microscopy (SEM) was used to capture high-resolution images of the sample solutions after pretreatment. The samples were sputter-coated with gold prior to imaging.

#### 2.6.5 Transmission electron microscopy analysis of the cell wall

Changes in the cell wall of ED-23G *A. fumigatus* containing 2 MIC AgNPs, 2 MIC Chi-AgNPs or no drug were observed via transmission electron microscopy. After incubation for 36 hours, the mycelium was fixed at 4°C with 2.5% sodium glutaraldehyde phosphate buffer. The sample was subsequently treated with a 1% osmic acid solution for 1–2 hours. The sample was then placed in 0.1 M phosphate buffer at pH 7.0 and rinsed three times for 15 minutes each. The samples were then dehydrated with ethanol solutions of different concentrations for 15 minutes each and finally dehydrated with 100% ethanol twice for 20 minutes each. The samples were then soaked in pure acetone for 20 minutes. Next, the sample was treated with a mixture of the encapsulation agent and acetone and underwent a combination treatment of the embedding agent and acetone. The permeated sample was embedded and heated overnight at 70°C to complete curing. The cured sample was then sliced via an ultrathin microtome, resulting in slices between 70 and 90 nm thick. Finally, the slices were stained with uranyl acetate and lead citrate and examined via transmission electron microscopy at an accelerating voltage of 80 kV (Sun et al., 2022a).

### 2.7 Determination of antifungal activity

#### 2.7.1 Minimum inhibitory concentration test

The MIC was determined via a method established by the Institute of Clinical and Laboratory Standards (CLSI document M38-A2) (Espinel-Ingroff et al., 2011). We used minimal (MM) liquid medium instead of RPMI-1640 medium to prepare several centrifuge tubes and obtained a solution of chitosan at different concentrations. These MM media had no effect on the test results (Du et al., 2023). Then, 20 μL of fungal mixture diluted to 1×10^6^ CFU/mL was inoculated in a centrifuge tube and incubated in an incubator at 37°C for 36 hours. The MIC of the Chi-AgNPs against *A. fumigatus* was determined by comparing the turbidity of each tube liquid. The minimum concentration of Chi-AgNPs in the MM without fungal growth (clear) was the MIC of the Chi-AgNPs. The MICs of chitosan and AgNPs against *A. fumigatus* were determined via the methods described above.

#### 2.7.2 Minimum fungicidal concentration test

Five kinds of *A. fumigatus* suspensions were diluted with MM liquid medium to a concentration of × 10^6^ CFU/mL, and three kinds of drug dilution methods were used to dilute the suspensions to 512, 256, 128, and 64 μg/mL. Three kinds of diluents were added to 100 μL in each well of a sterile 96-well plate, and five kinds of *A. fumigatus* fumigus suspensions were added to 100 μL. The mixture was incubated at 37°C for 24 h, 100 μL of liquid was added to the surface of a yeast agar (YAG) solid medium plate, and a sterilized coating was evenly applied to the surface of the medium 3 times according to the reference with adjustment (Franconi and Lupetti, 2023). During each application, to ensure an even distribution of the fungal mixture, the plate was rotated by 60°. After three applications were completed, one more application was applied around the edge of the plate to ensure that the edge area was covered by the fungal mixture. After application, the dish was covered tightly and allowed to dry at room temperature for 5 minutes. Finally, the Petri dishes were cultured in a constant-temperature incubator at 37°C for 36 hours. The number of colonies in the Petri dish with different drug concentrations was observed, and the minimum concentration of fewer than 5 colonies was the MFC.

### 2.8 Microbial growth curve

*A. fumigatus* strain ED-23G suspension was diluted with the MM liquid mixture to a concentration of 1×10^6^ CFU/mL. The three drug dilutions were diluted to 32 μL/mL. Then, 100 μL of the three kinds of diluted liquid was added to each well of a sterile 96-well plate, and 100 μL of the five kinds of *A. fumigatus* suspensions was added. The samples were incubated at 37°C in a constant-temperature incubator, and the optical density (OD) values were measured every 2 h with a 620 nm enzyme marker. The growth curve experiment was conducted with three independent replicates (n=3), and the data are presented as the mean OD620 ± SD.

### 2.9 *In vivo* antifungal effect

*A. fumigatus* strain ED-23G suspension was diluted with MM liquid medium to 1×10^6^ CFU/mL. The three drug dilutions were diluted to 16 μg/mL. *G. mellonella* larvae of similar size and weight (250–350 mg) were selected for the experiment. The samples were divided into eight groups, with 10 *G. mellonella* larvae per group. In the normal saline group, 20 μL of normal saline was injected into the second right group with a microsyringe. In the drug treatment groups, 10 μL of fungal mixture was injected, and then, 10 μL of diluted chitosan and AgNPs and Chi-AgNPs were injected 1 h later. Larvae were observed every 24 h, and death was defined as lack of movement upon stimulation (Sun et al., 2022b). The survival experiment was performed three times independently with consistent results, and the data from one representative experiment (n=10 larvae per group) are shown. Survival curves were plotted via the Kaplan-Meier method, and statistical comparisons between groups were analyzed by the log-rank test.

### 2.10 Reactive oxygen species measurement

To assess the induction of ROS in *A. fumigatus* strain ED-23G, the fungal suspension was first diluted in MM liquid medium to a concentration of 1x10^6^ CFU/mL. One hundred microliters of this diluted suspension were distributed into each well of a black, transparent-bottomed, sterile 96-well plate and incubated at 37°C for 12 hours to allow the mycelia to grow. The test drugs were prepared by diluting them in sterile PBS to a concentration of 8 μg/mL. After the 12 h incubation period, the mycelial suspension was aspirated from the wells, the mycelia were washed twice with sterile PBS to remove residual nutrients and debris, and the diluted drugs were added. The plate was returned to the incubator at 37°C for an additional 3 hours. Following the 3-h drug treatment, the drug mixture was aspirated, and the mycelia were further washed twice with sterile PBS. H_2_DCFH-DA (2’,7’-dichlorofluorescin diacetate) was added to each well to a final concentration of 40 μg/mL, ensuring that the addition and subsequent incubation steps were performed in the dark to prevent photobleaching. The plate was incubated at 37°C in the dark for 1 hour to allow H_2_DCFH-DA to enter the cells and be oxidized by ROS into a fluorescent product. Finally, a multifunctional microplate reader was used to measure the emission fluorescence intensity at a wavelength of 525 nm under excitation at 488 nm (Engelbrecht et al., 2024). The fluorescence intensity is proportional to the level of ROS present in the drug-treated mycelia. The data are expressed as the mean fluorescence intensity ± SD. Statistical significance was determined via one-way ANOVA followed by Tukey’s *post-hoc* test.

### 2.11 Experimental replicates and statistical analysis

All biological assays were performed with freshly prepared nanocomposites to ensure consistency. All *in vitro* and *in vivo* experiments were performed with three independent biological replicates (n ≥ 3), each conducted on separate days with freshly prepared reagents and fungal cultures, unless otherwise specified. The data are presented as the means ± standard deviations (SDs). Statistical analyses were performed via GraphPad Prism software (version 10.2.3). Differences were considered statistically significant at *p* < 0.05.

## 3 Results

### 3.1 Characterization of chitosan, AgNPs, and Chi-AgNPs

**Figure 1:**
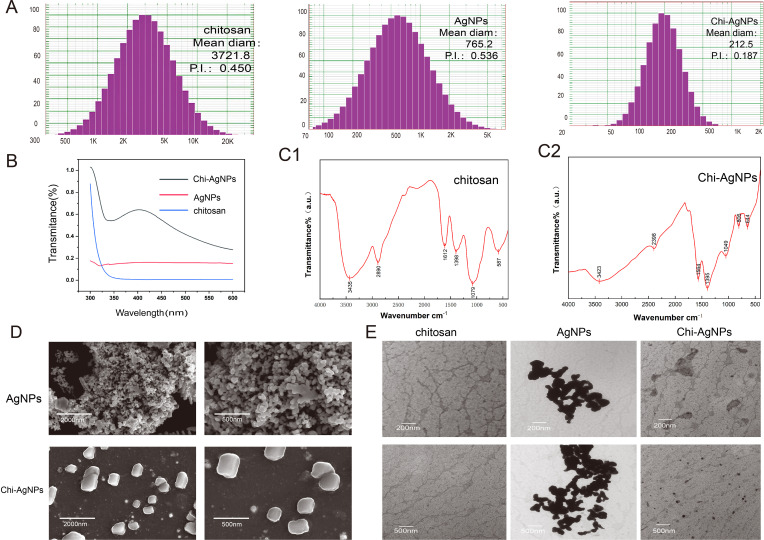
Characterization of chitosan, AgNPs and Chi-AgNPs: **(A)** Particle size analysis of the nanocomposites. **(B)** Ultraviolet absorption spectra of the nanocomposites; **(C)** Fourier infrared absorption spectra of the nanocomposites; **(C1)** FTIR spectra of chitosan; **(C2)** FTIR spectra of Chi-AgNPs **(D)** Nanocomposite scanning electron microscope. Morphology of AgNPs and Chi-AgNPs at different magnifications. **(E)** Nanocomposite transmission electron microscopy image showing the chitosan, AgNPs and Chi-AgNPs morphology at different magnifications.

In this study, the particle sizes of chitosan, AgNPs, and Chi-AgNPs were measured via a particle analyzer. The results revealed that the particle diameter of chitosan ranged from approximately 500 nm to 12,000 nm, with an average diameter of 3721.8 nm. Owing to their poor dispersibility, AgNPs easily aggregate, resulting in measured particle sizes ranging from approximately 100 nm to 4000 nm, with an average diameter of 765.2 nm. In contrast, the particle size of the Chi-AgNPs ranged from 70 nm to 600 nm, with an average diameter of 212.5 nm. The dispersion index (P.I.) of Chi-Ag was 0.187, whereas it was 0.45 for Chi and 0.536 for AgNPs, indicating that the Chi-AgNPs dispersion system was more stable and uniform based on immediate characterization data (**Figure 1A**).

UV–visible spectroscopy was used to characterize the three substances, and the results were as follows: the optical density (OD) spectrum of chitosani showed high transmittance at lower wavelengths (~300 nm), with transmittance rapidly decreasing as the wavelength increased, reaching zero transmittance above 350 nm. Pure chitosan lacked any significant absorption peaks in this wavelength range, suggesting the absence of chemical groups in its molecular structure that resonate with and absorb light at these specific wavelengths. AgNPs exhibit relatively constant low transmittance across the entire wavelength range without notable peaks, which is characteristic of silver nanoparticles absorbing light broadly due to their surface plasmon resonance (SPR). However, Chi-AgNPs displayed a complex spectrum with several notable features: high transmittance at lower wavelengths (~300 nm), a local minimum at approximately 330 nm, a broad peak centered at approximately 400–420 nm, and a gradual decrease in transmittance at higher wavelengths. The broad peak in the Chi-AgNPs spectrum is attributed to the surface plasmon resonance (SPR) of the silver nanoparticles, which is modified by their interaction with chitosan. These unique features suggest the successful integration of silver nanoparticles with chitosan (**Figure 1B**).

FTIR spectroscopy was employed to analyze the functional groups in chitosan and Chi-AgNPs (**Figures 1**, **C1**, **C2**). In the FTIR spectrum of chitosan, the N-H and O-H stretching vibrations merged to form a broad peak at 3435 cm⁻¹ (amide A). The characteristic peak at 2890 cm⁻¹ was assigned to the stretching vibration of the fatty CH bond (amide B). The absorption peaks at 1612 cm⁻¹, 1398 cm⁻¹, and 1079 cm⁻¹ were attributed to N–H bending (amide I), CH bending (amide II), and C–O stretching vibrations (amide III), respectively. In the FTIR spectrum of Chi-AgNPs, the peak at 3435 cm⁻¹ redshifted to 3423 cm⁻¹, indicating that the amino and hydroxyl groups participated in silver chelation via coordination bonds. Additionally, the amide I, II, and III peaks shifted to 1564 cm⁻¹, 1395 cm⁻¹, and 1049 cm⁻¹, respectively, with reduced intensities, further confirming the interaction between the AgNPs and the amino/hydroxyl groups of chitosan.

The three-dimensional structures of chitosan, AgNPs, and Chi-AgNPs were analyzed via SEM. As shown in **Figure 1D**, the AgNPs exhibited poor dispersion, a tendency to aggregate, and large particle sizes. In contrast, the Chi-AgNPs particles were approximately 200 nm in size, which was consistent with the measured particle size, and presented a transparent chitosan coating on their surface (**Figure 1D**). Compared with the AgNPs dispersions, the Chi-AgNPs dispersions were more uniform, with the particles exhibiting a spherical and consistent morphology.

TEM provided additional information on the particle shape, size, and distribution. As shown in **Figure 1E**, chitosan dissolved in water formed irregular sheet-like structures, which is consistent with its measured size range of 500–12000 nm. As shown in **Figure 1E**, the AgNPs aggregated into irregular clumps, corroborating the SEM findings that such aggregation reduces their antimicrobial efficacy. In contrast, the Chi-AgNPs particles appeared dark and nearly spherical with a relatively uniform distribution (**Figure 1E**), further confirming their improved stability and morphology.

### 3.2 Antimicrobial effects and *in vitro* antifungal properties of chitosan, AgNPs, and Chi-AgNPs against *A. fumigatus*

**Figure 2:**
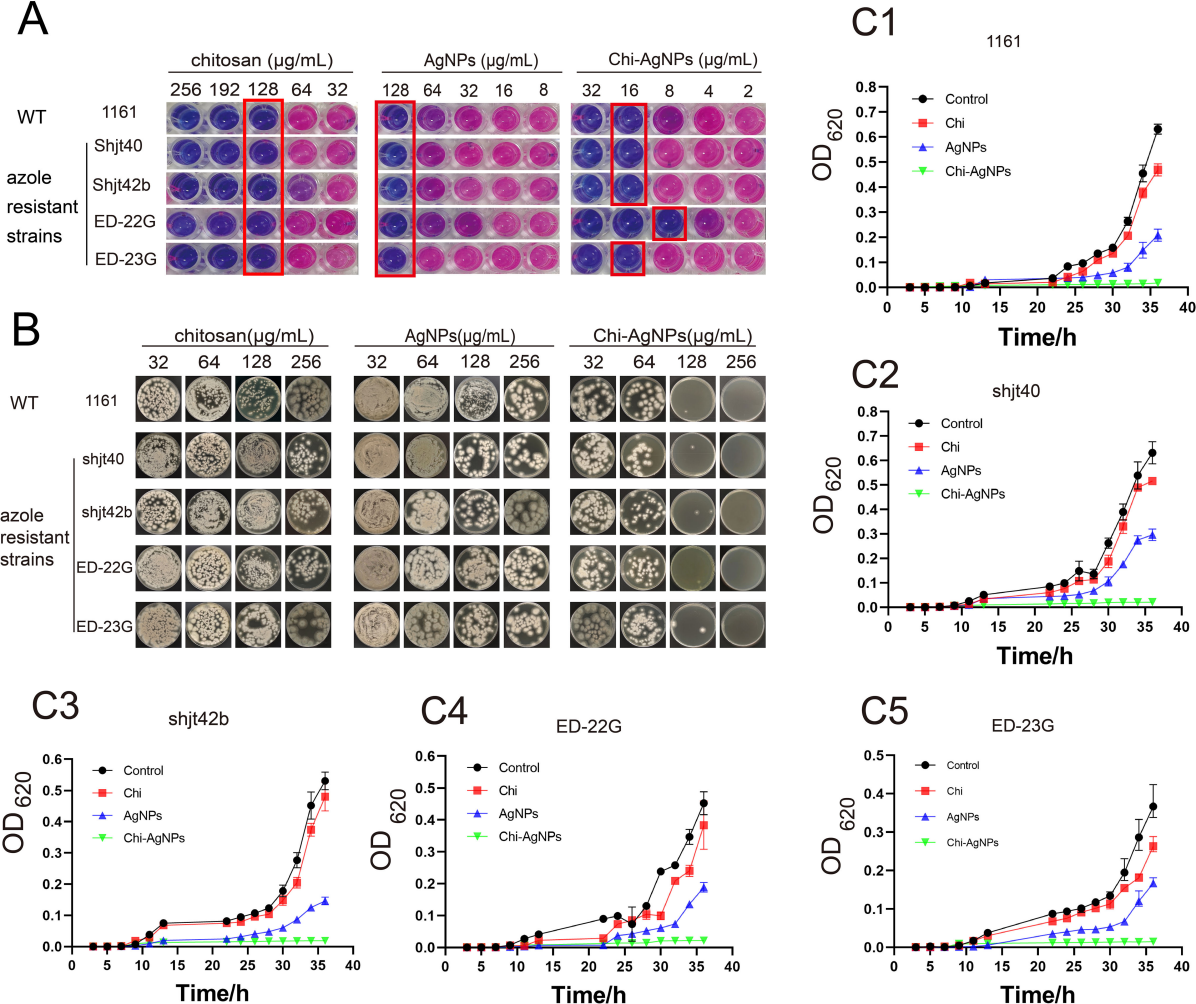
*In vitro* antifungal properties of chitosan, AgNPs, and Chi-AgNPs against *A. fumigatus*. **(A)** MICs of chitosan, AgNPs, and Chi-AgNPs against five strains of *A*. *fumigatus*. In each group of pictures, medium blue represents no metabolic activity, and pink purple represents high metabolic activity, that is, a high fungal content. **(B)** MFCs of chitosan, AgNPs, and Chi-AgNPs against five strains of *A*. *fumigatus.***(C)** Fungal growth curve; **(C1–C5)** are the OD values of five fungi treated with the same concentration of three different drugs within 36 hours.

The antimicrobial efficacy of chitosan, AgNPs, and Chi-AgNPs against *A. fumigatus* was evaluated via resazurin staining. The blue media indicated no metabolic activity, whereas the pink/purple media indicated high metabolic activity and fungal growth. The MIC of Chi-AgNPs against the wild-type *A. fumigatus* strain (1161) and three azole-resistant strains (shjt 40, shjt 42b and ED-23G) was 16 μg/mL, whereas its MIC against another azole-resistant strain (ED-22G) was 8 μg/mL. In contrast, the MICs of chitosan and AgNPs were 128 μg/mL for all strains (**Figure 2A**, **Table 1**). These results indicate that Chi-AgNPs exhibit significantly better antimicrobial efficacy than chitosan or AgNPs alone.

**Table 1:** Drug susceptibility test of itraconazole, chitosan, AgNPs, and Chi-AgNPs.

Strains	MIC (μg/mL)				MFC (μg/mL)		
	Itraconazole	Chitosan	AgNPs	Chi-AgNPs	Chitosan	AgNPs	Chi-AgNPs
1161	0.5	128	128	16	>256	>256	128
Shjt40	4	128	128	16	>256	>256	128
Shjt42b	>8	128	128	16	>256	>256	128
ED-22G	>16	128	128	8	>256	>256	128
ED-23G	2	128	128	16	>256	>256	128

Furthermore, when fungal colonies were plated with 128 μg/mL Chi-AgNPs, fewer than five colonies were observed, whereas the minimum fungicidal concentrations (MFCs) of chitosan and AgNPs were both greater than 256 μg/mL. This highlights the superior fungicidal activity of Chi-AgNPs (**Figure 2B**, **Table 1**). The growth curves of *A. fumigatus* strains treated with 16 μg/mL chitosan, AgNPs, or Chi-AgNPs were also examined. Chi-AgNPs had a greater inhibitory effect on fungal growth, as evidenced by lower OD620 values (**Figure 2C**).

### 3.3 *In vivo* antimicrobial properties and toxicity in an invertebrate model of chitosan, AgNPs and Chi-AgNPs against *A. fumigatus*

**Figure 3:**
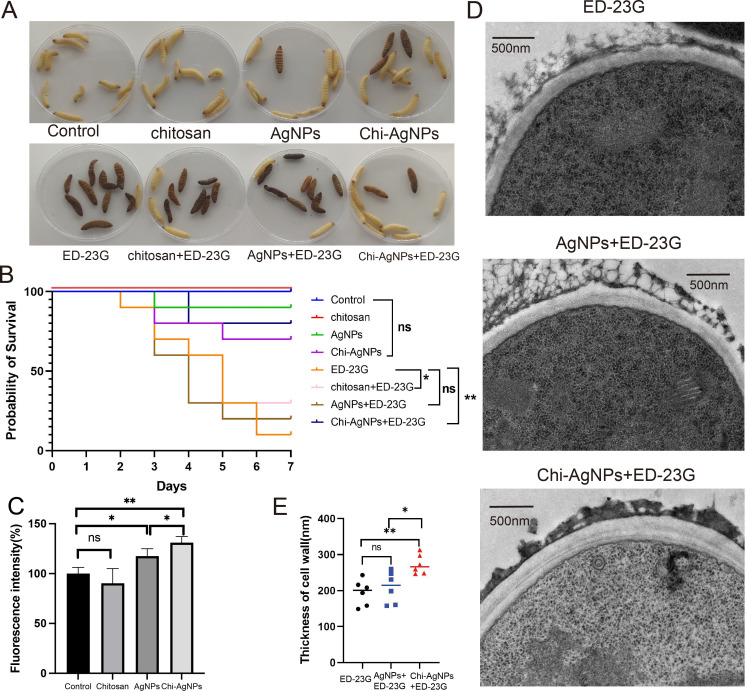
*In vivo* antimicrobial properties and toxicity in an invertebrate model of chitosan, AgNPs and Chi-AgNPs against *A. fumigatus*. **(A)** On day 7 of the *in vivo* experiment using *G. mellonella* larvae, the following groups were observed: saline control, chitosan alone, AgNPs alone, Chi‑AgNPs alone, *A. fumigatus* ED‑23G alone, and ED‑23G combined with chitosan, AgNPs, or Chi‑AgNPs. Dead larvae appeared dark brown, whereas surviving larvae remained white. **(B)** Survival curve of *G. mellonella* larvae (n=10 per group). *p < 0.05; **p < 0.01; ns, *p* > 0.05 according to log rank analysis. **(C)** Intracellular ROS levels in *A*. *fumigatus* (ED-23G) hyphae after treatment with chitosan, AgNPs, or Chi-AgNPs for 3 h. Data are presented as mean fluorescence intensity ± SD from three independent experiments (n=3). Significance was determined by one-way ANOVA with Tukey’s *post-hoc* test. *p < 0.05, **p < 0.01, ns: *p* > 0.05. **(D)** Transmission electron microscopy (TEM) of *A*. *fumigatus* without or with AgNPs or with Chi-AgNPs treatment. Scale bar = 500 nm. **(E)** Cell wall thickness of *A*. *fumigatus* without or with AgNPs or with Chi-AgNPs treatment. Data are shown as mean ± SD (n = 6). *p < 0.05; **p < 0.01; ns, *p* > 0.05 according to one-way ANOVA with Tukey’s *post-hoc* test.

The *in vivo* antifungal activity of chitosan, AgNPs, and Chi-AgNPs was assessed in *G. mellonella* larvae by injecting saline, drug solutions, fungal suspensions, or combinations. As shown in **Figure 3A**, no larval deaths occurred in the saline or chitosan groups, whereas one larva died in the AgNPs group. In the fungal-only group, nine larvae died. Among the treatment groups, the Chi-AgNPs group presented the lowest mortality (two deaths), whereas the chitosan and AgNPs groups presented seven and eight deaths, respectively. These results indicate that Chi-AgNPs demonstrate superior antifungal activity with negligible toxicity, as there was no significant difference between the three drug groups and the saline control group (**Figure 3B**).

Reactive oxygen species (ROS) levels were measured to investigate the mechanism of fungal inhibition. Chi-AgNPs treatment induced the production of large amounts of ROS, which likely damaged fungal cell membranes and led to fungal death. Compared with Chi-AgNPs treatment, AgNPs treatment induced a smaller amount of ROS, which may explain the reduced antimicrobial efficacy of AgNPs (**Figure 3C**). TEM imaging of fungal cell walls revealed that compared with AgNPs treatment, Chi-AgNPs treatment was associated with more apparent morphological alterations, such as deformation, loosening, and thickening of the cell walls (**Figures 3D, E**). These observations, coupled with the elevated ROS levels (**Figure 3C**), suggest a potential mechanism underlying the superior antifungal efficacy of Chi-AgNPs.

## 4 Discussion

**Figure 4:**
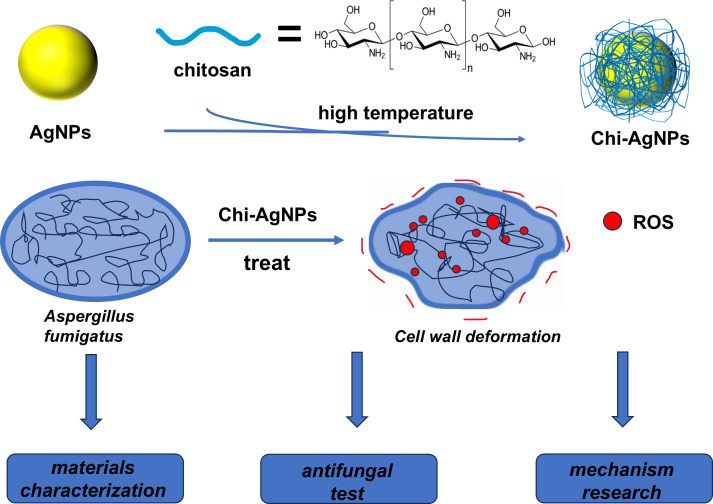
Chitosan and AgNPs were used to synthesize antimicrobial materials: Chi-AgNPs, which focused on material characterization, antifungal test and mechanism research. Chi-AgNPs induce ROS burst and cell wall deformation, leading to fungal death.

This study provides a systematic evaluation of chitosan-silver nanocomposites (Chi-AgNPs) as a potent antifungal agent against azole-resistant *A. fumigatus*. As shown in **Figure 4**, through a combination of particle size analysis, UV–visible spectroscopy, FTIR, SEM, and TEM, Our key findings demonstrate that Chi-AgNPs possess: (1) superior physicochemical properties, including smaller size, uniform distribution, and enhanced stability compared to chitosan or AgNPs alone; (2) significantly greater *in vitro* and *in vivo* antifungal efficacy against both wild-type and azole-resistant strains; and (3) a mechanism of action involving enhanced ROS generation and consequential fungal cell wall damage. These results collectively underscore the potential of Chi-AgNPs as a promising alternative to combat resistant fungal infections.

The enhanced performance of Chi-AgNPs is fundamentally linked to their improved physicochemical characteristics. Our particle size analysis revealed that Chi-AgNPs were significantly smaller and more uniform (average diameter: 212.5 nm) than chitosan (3721.8 nm) or AgNPs (765.2 nm). The lower dispersion index (P.I.) of Chi-AgNPs (0.187) compared to its individual components indicates a more stable colloidal system, a critical factor for biological applications. This observation aligns with previous reports that chitosan acts as an effective stabilizer, preventing the aggregation of silver nanoparticles and thereby improving their dispersibility and bioavailability (Dubey et al., 2023; Yahya and Alharbi, 2023). The ability of Chi-AgNPs to maintain a consistent particle size is critical for their enhanced biological activity, as smaller, uniformly distributed nanoparticles exhibit better surface interactions with microbial cells (Visinescu et al., 2018). It is noteworthy that the chemical reduction method employed in this study was chosen to ensure precise control over particle size, uniformity, and batch-to-batch reproducibility—parameters that are crucial for reliable comparative mechanistic studies. While green synthesis approaches offer advantages in biocompatibility and environmental friendliness, our method prioritized the generation of consistent and well-characterized nanocomposites, which is essential for establishing clear structure-activity relationships and evaluating antifungal efficacy in a controlled manner.

The successful integration of silver nanoparticles with chitosan was further confirmed by UV-Vis spectroscopy, which showed a characteristic SPR peak at 400–420 nm for Chi-AgNPs, and by FTIR analysis, which indicated a redshift in the N-H/O-H stretching vibration (from 3435 cm⁻¹ to 3423 cm⁻¹), suggesting coordination between the amino/hydroxyl groups of chitosan and AgNPs (Zhang et al., 2025). Morphological analyses via SEM and TEM corroborated these findings, showing that while AgNPs alone formed large, irregular aggregates, Chi-AgNPs were spherical, uniformly distributed, and coated with a chitosan layer. This refined morphology likely facilitates better interaction with fungal cells, enhancing antimicrobial activity (Rasheed et al., 2023).

The superior physicochemical properties of Chi-AgNPs directly translated into markedly improved antifungal efficacy *in vitro*. The MIC of Chi-AgNPs (8–16 μg/mL) against various *A. fumigatus* strains was substantially lower than that of chitosan or AgNPs alone (128 μg/mL). More importantly, Chi-AgNPs exhibited potent fungicidal activity, with an MFC of 128 μg/mL, whereas the MFC for both chitosan and AgNPs exceeded 256 μg/mL. This represents an 8- to 16-fold enhancement in inhibitory activity and at least a 2-fold improvement in fungicidal activity, highlighting a clear synergistic effect between chitosan and AgNPs. This synergy is consistent with prior studies on chitosan-silver composites against other pathogens (Kulatunga et al., 2017). Notably, the MIC values of our Chi-AgNPs (8-16 μg/mL) are lower than those reported for similar nanocomposites against other fungi, such as a chitosan-silver nanocomposite (CAgNC) against *Candida albicans* (MIC: 25 μg/mL) (Kulatunga et al., 2017), underscoring the potent antifungal profile of our formulation. Moreover, our work specifically demonstrates this enhanced synergistic effect against clinically relevant azole-resistant *A. fumigatus*. The growth curve analysis confirmed the sustained inhibitory effect of Chi-AgNPs, as evidenced by the significantly lower OD620 values over time compared to treatments with individual components.

To elucidate the mechanism behind the superior efficacy of Chi-AgNPs, we investigated their ability to induce ROS and cause cellular damage. Our results indicate that Chi-AgNPs triggered a significantly higher level of intracellular ROS in *A. fumigatus* hyphae compared to AgNPs alone. The enhanced ROS generation observed with Chi-AgNPs may be attributed to the synergistic interaction between chitosan and silver nanoparticles. Chitosan improves the dispersion and cellular uptake of AgNPs, facilitating greater intracellular release of Ag^+^ ions. Additionally, chitosan itself can disrupt fungal membrane integrity, sensitizing the cells to silver-induced oxidative stress and leading to amplified ROS production. ROS generation is a well-documented antimicrobial mechanism of silver nanoparticles, leading to oxidative stress, lipid peroxidation, and damage to proteins and DNA (Liang et al., 2023; Godakhindi et al., 2025). The enhanced ROS production observed with Chi-AgNPs suggests that the interaction between chitosan and silver nanoparticles may amplify oxidative stress in fungal cells, which could contribute to cell damage. In line with these findings, TEM imaging of *A. fumigatus* treated with Chi-AgNPs revealed apparent morphological alterations, including deformation, loosening, and thickening of the fungal cell wall, providing visual support for this hypothesis.

These findings are consistent with recent studies that highlighted the efficacy of chitosan–silver nanocomposites against fungal pathogens. For example, Dananjaya et al. (2017) demonstrated that chitosan-silver composites exhibited enhanced antifungal activity against *Fusarium oxysporum* due to synergistic ROS generation and cell wall targeting. Similarly, Mondéjar-López et al. (2023) reported that silver nanoparticles functionalized with chitosan showed superior activity against fungal strains compared with unmodified silver nanoparticles.

The *in vivo* efficacy and toxicity in an invertebrate model of Chi-AgNPs were validated using the *G. mellonella* infection model. In larvae infected with a lethal dose of azole-resistant *A. fumigatus*, treatment with Chi-AgNPs resulted in the highest survival rate, significantly outperforming treatments with chitosan or AgNPs alone. Crucially, the Chi-AgNPs treatment group showed no significant difference in mortality compared to the saline control group, indicating negligible inherent toxicity of the nanocomposite at the therapeutic dose. This superior *in vivo* performance, coupled with low toxicity in an invertebrate model, is consistent with the known safety profile of chitosan (Rasheed et al., 2023) and positions Chi-AgNPs as a highly promising candidate for further therapeutic development.

Based on our findings, the mode of action of Chi-AgNPs can be summarized as follows: *In vitro*, Chi-AgNPs exert their antifungal activity primarily through enhanced ROS generation, which leads to oxidative damage of cellular components, and through direct disruption of the fungal cell wall, as evidenced by TEM imaging (**Figures 3D, E**). The synergistic interaction between chitosan and silver nanoparticles facilitates greater cellular uptake and Ag^+^ release, amplifying ROS production and compromising membrane integrity. *In vivo*, in the *G. mellonella* infection model, Chi-AgNPs demonstrated negligible systemic toxicity, as reflected by the high survival rate of Chi-AgNPs-treated larvae (**Figures 3A, B**). The low mortality observed in the Chi-AgNPs group, comparable to the saline control, suggests that the nanocomposite does not elicit significant host toxicity at therapeutic concentrations, likely due to the biocompatible and biodegradable nature of chitosan, which mitigates the potential cytotoxic effects of silver nanoparticles.

While previous studies have focused primarily on bacterial pathogens, our work emphasizes the importance of Chi-AgNPs in combating fungal infections, particularly those caused by azole-resistant *A. fumigatus*. This is a critical area of research, as fungal infections are increasingly recognized as a significant threat to public health (Fisher et al., 2022). Therefore, the novelty of this study is that direct comparison of Chi-AgNPs against azole-resistant *A. fumigatus* strains and demonstration of synergistic ROS induction and cell wall damage.

While this study provides robust evidence for the potential of Chi-AgNPs, certain limitations should be acknowledged. Firstly, a direct comparison with first-line azole drugs or echinocandins was not within the scope of this initial characterization study; such comparisons will be essential in future work to fully contextualize the potency of Chi-AgNPs. Secondly, the precise molecular pathways leading to the enhanced ROS generation and the specific interactions between the nanocomposite and fungal cell wall components warrant further investigation using techniques such as transcriptomics or proteomics. Future translational studies should focus on dose optimization and safety evaluation in mammalian models to further establish the therapeutic window.

## 5 Conclusions

In conclusion, this study demonstrates that the Chi-AgNPs exhibits significantly enhanced antifungal activity against azole-resistant *A. fumigatus* compared to its individual components. This superiority is underpinned by improved nanoparticle stability and morphology, a synergistic combination of chitosan’s membrane-targeting action and silver’s ROS-generating capability, leading to severe fungal cell wall damage. Our findings provide a strong foundation for the development of Chi-AgNPs as a viable and effective strategy to address the growing challenge of antifungal resistance.

## Data Availability

The original contributions presented in the study are included in the article/supplementary material. Further inquiries can be directed to the corresponding author.
